# Multi-Omics Approaches: The Key to Improving Respiratory Health in People With Cystic Fibrosis?

**DOI:** 10.3389/fphar.2020.569821

**Published:** 2020-09-03

**Authors:** Andrew J. Lee, Gisli G. Einarsson, Deirdre F. Gilpin, Michael M. Tunney

**Affiliations:** ^1^ Halo Research Group, Queen’s University Belfast, Belfast, United Kingdom; ^2^ Centre for Experimental Medicine, School of Medicine, Dentistry and Biomedical Sciences, Queen’s University Belfast, Belfast, United Kingdom; ^3^ School of Pharmacy, Queen’s University Belfast, Belfast, United Kingdom

**Keywords:** cystic fibrosis, inflammation, lungs, metagenomics, microbiome, microbiota, multi-omics

## Abstract

The advent of high-throughput multi-omics technologies has underpinned the expansion in lung microbiome research, increasing our understanding of the nature, complexity and significance of the polymicrobial communities harbored by people with CF (PWCF). Having established that structurally complex microbial communities exist within the airways, the focus of recent research has now widened to investigating the function and dynamics of the resident microbiota during disease as well as in health. With further refinement, multi-omics approaches present the opportunity to untangle the complex interplay between microbe–microbe and microbe–host interactions in the lung and the relationship with respiratory disease progression, offering invaluable opportunities to discover new therapeutic approaches for our management of airway infection in CF.

## Introduction

In recent years, the growing and widespread application of genomics focused, culture-independent techniques for microbiological analysis has been universally acknowledged as revolutionary for the research and management of disease (Malla et al., 2019). As the field of genomics has matured, we have learned that healthy lungs are inhabited by a diverse and complex array of airborne particles and microbial life (Hilty et al., 2010; Dickson and Huffnagle, 2015) . Moreover, we have realized the extent and significance of the microbiome and its role in respiratory health and disease (Nguyen et al., 2015; Barcik et al., 2020).

Technological advances have enabled cost-efficient, high-throughput, in-depth analysis of transcripts, proteins, and metabolites (e.g. metatranscriptomics, metaproteomics, metabolomics, and metabologenomics) exponentially increasing our understanding of our own microbiota, the vital role it plays in maintaining our health and offering opportunities to understand its capacity to contribute to disease (**Table 1**).

**Table 1:** Definitions of main terms and nomenclature.

Term	Definition	References
**Dysbiosis**	Non-homeostatic imbalance and loss of diversity in the resident microbial community often associated with disease states	(Petersen and Round, 2014)
**Microbiota**	All the microorganisms inhabiting a specific niche	(Lederberg, 2000; Turnbaugh et al., 2007)
**Microbiome**	Collective genetic material of all microorganisms and particles (bacteria, viruses, fungi, protozoa) present in a community	(Lederberg, 2000; Turnbaugh et al., 2007)
**Bacteriome**	Collection of all the bacterial genomes present within a community	
**Mycobiome**	Collection of all the fungal genomes present within a community	
**Virome**	Collection of all the viral genomes present within a community	
**Resistome**	Collection of all the genes and genetic precursors of anti-microbial resistance, found in both pathogenic and non-pathogenic bacteria	(Wright, 2007)
**Next-generation sequencing (NGS)**	High-throughput technologies facilitating rapid, parallel and cost-effective DNA sequencing	
**Metagenomics**	Functional analysis of all the collective genomes obtained from every individual member of the microbial community, including archaea, viruses and fungi	(Tringe and Rubin, 2005)
**Metabolomics**	Analysis of the complete set of metabolites from all processes present in a population	(Siggins et al., 2012; Aguiar-Pulido et al., 2016)
**Metatranscriptomics**	Study of microbial gene expression within habitats, providing insights on what genes are functionally active in a community	(Aguiar-Pulido et al., 2016)
**Metabologenomics**	Study, identification and correlation of microbial gene clusters responsible for the biosynthesis of expressed metabolites	(Goering et al., 2016)
**Marker Gene Analysis (MGA)**	Amplicon based culture-independent method often used for microbial classification by targeting 16S, 23S or 18S rRNA genes	
**Whole Genome Shotgun Sequencing (WGSS)**	Culture-independent genome wide NGS approach used for gene cataloging and functional inference	
**Whole Genome Sequencing (WGS)**	Culture-dependent NGS of whole cells, low throughput but provides practical data to improve metagenomics	
**Operational Taxonomic Unit (OTU)**	Clusters of related sequences within a percent sequence similarity threshold (>97% similarity), proxy for species-level divergence	
**Multi-omics**	Assimilation of data from various “omics” technologies such as microbiomics, metagenomics, metatranscriptomics, metaproteomics, and metabolomics	(Vilanova and Porcar, 2016)
**Genome Mining**	Computationally intensive process of exploiting genomic information to isolate, characterize and experimentally verify products of potentially useful genes	(Corre and Challis, 2010)
**Prebiotics**	A substrate that is selectively utilized by host microorganisms conferring a health benefit	(Gibson et al., 2017)
**Probiotics**	Live microorganisms which when consumed in adequate amounts confer a health benefit on the host	(Food and Agriculture Organization and World Health Organization Expert Consultation, 2001)

Collectively the respiratory microbiome consists of the upper (nasal and oral passages) and lower (lungs) airways. In those without respiratory disease, specific differences in the composition and load of the microbiota between these connected mucosal sites are apparent (**Table 2**). No firm consensus exists regarding the bacterial composition of a “typical healthy” lung; however it is now accepted that the lower airways harbor a diverse and dynamic ecosystem inhabited by a range of facultatively and obligately aerobic and anaerobic microorganisms with considerably greater inter versus intrasubject variation in composition (Erb-Downward et al., 2011; Invernizzi et al., 2020).

**Table 2:** Bacterial composition of the “healthy” airways.

Microbiome	Predominant Members	References
**Nasal**	**Phyla:** Actinobacteria, (principally families *Corynebacteriaceae* and *Propionibacteriaceae*), Bacteroidetes, Firmicutes, Proteobacteria **Genera:** *Bifidobacterium, Corynebacterium, Staphylococcus, Streptococcus, Dolosigranulum, Moraxella*	(Lemon et al., 2010; Bassis et al., 2014; de Steenhuijsen Piters et al., 2015; Mammen and Sethi, 2016; Kumpitsch et al., 2019; Dimitri-Pinheiro et al., 2020)
**Oral**	**Phyla:** Actinobacteria (families *Actinomycetaceae* and *Micrococcaceae*), Bacteroidetes, Chlamydiae, Chloroflexi, Firmicutes, Fusobacteria, Gracilibacteria (formerly GN02), Proteobacteria, Spirochaetes, SR1 (candidate phylum Absconditabacteria), Synergistetes, Saccharibacteria (formerly TM7) **Genera (selected representatives):** *Actinomyces, Campylobacter, Corynebacteria, Fusobacterium,Haemophilus, Lactobacillus Moraxella, Neisseria, Prevotella, Rothia, Veillonella, Streptococcus*	(Bassis et al., 2014; McLean, 2014; Ramakrishnan et al., 2016; Huffnagle et al., 2017; Sharma et al., 2018; Sultan et al., 2018; Deo and Deshmukh, 2019)
**Lung**	**Phyla:** Actinobacteria, Bacteroidetes, Firmicutes, Fusobacteria, Proteobacteria **Genera:** *Fusobacterium, Haemophilus, Neisseria, Prevotella, Streptococcus, Veillonella*	(Tunney et al., 2008; Hilty et al., 2010; Charlson et al., 2011; Fodor et al., 2012; Segal et al., 2013; Einarsson et al., 2016; Moffatt and Cookson, 2017; Mendez et al., 2019)

## The Lung Microbiota During Disease

Personal health is linked to the presence of a broad and diverse microbiota, and this varies widely from individual to individual. Evidence thus far suggests that any disorder in the balance of the communities present, which ultimately leads to a loss of microbial diversity (dysbiosis) could potentially act as a catalyst for the development of illness (Malla et al., 2019). In CF, *Achromobacter*, *Burkholderia*, *Pseudomonas*, *Staphylococcus*, *Stenotrophomonas*, and *Streptococcus* spp. in the airways of PWCF has been described (Fodor et al., 2012; Feigelman et al., 2017; Frayman et al., 2017; Einarsson et al., 2019a). The presence of obligate anaerobes such as *Prevotella* and *Veillonella* in the CF lung has also been noted (Tunney et al., 2008; Field et al., 2010; Tunney et al., 2011) and such diverse taxa can potentially negatively or positively impact respiratory health depending on the species present (O’Neill et al., 2015; Sherrard et al., 2016). Moreover, it is worth noting that the prevalence of non-tuberculosus mycobacterial (NTM) respiratory infection in CF has been increasing in recent years, with those commonly isolated belonging to either *Mycobacterium avium* complex (MAC) or *Mycobacterium abscessus* group (MABS) (Salsgiver et al., 2016; Adjemian et al., 2018; Martiniano et al., 2019).

## Microbial Dysbiosis and the Gut–Lung Axis

**Figure 1:**
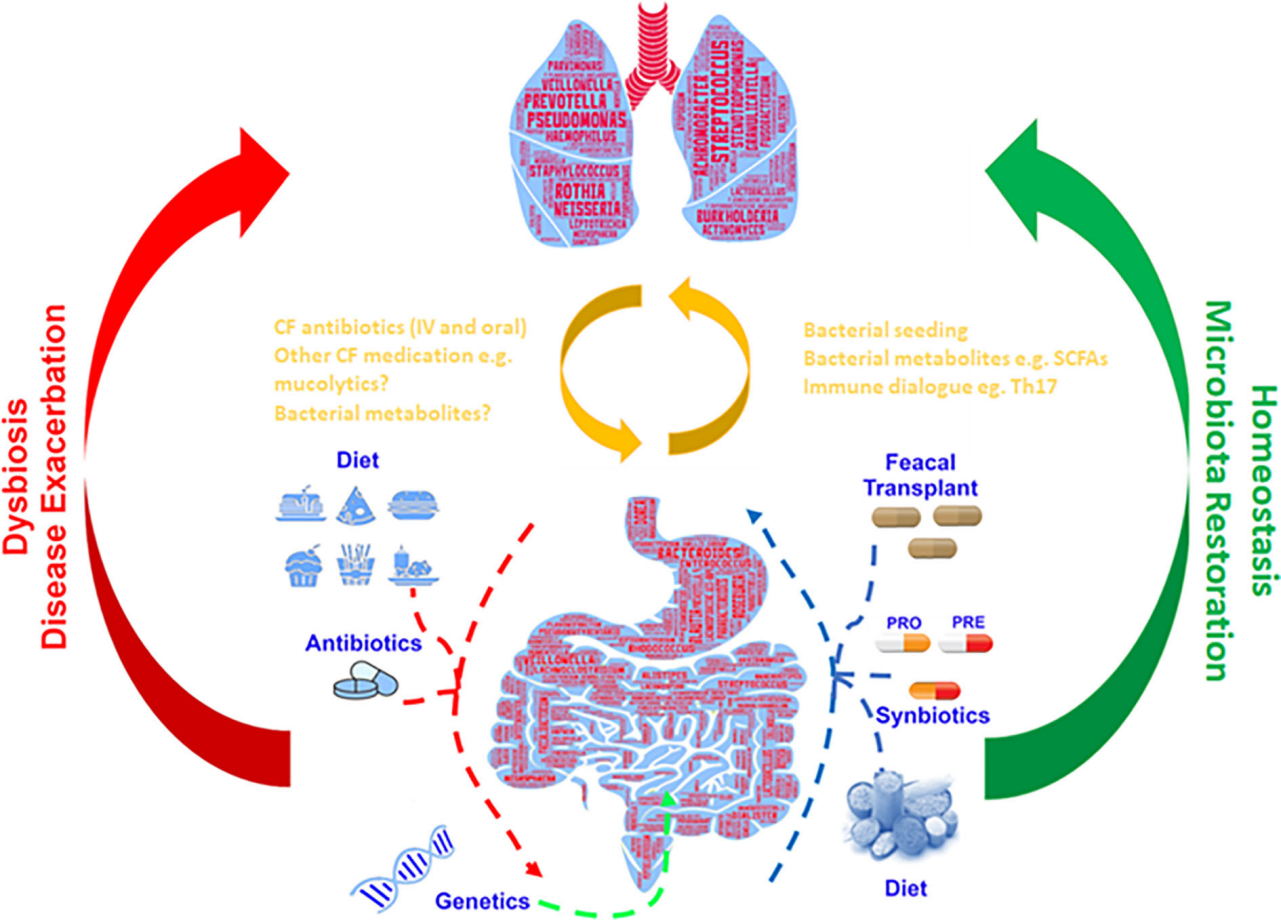
Gut–lung axis: Schematic depiction of the bidirectional cross-talk between the gut and lung which helps maintain microbial homeostasis. Respiratory disease, genetics and additional external factors can modulate the balance of this axis leading to the formation of a dysbiotic state.

Numerous studies have shown the importance of the gut microbiome in metabolic function (O’Hara and Shanahan, 2006), immune function (Cho and Blaser, 2012), pathogen resistance (Kamada et al., 2013) and chronic inflammation (Lobionda et al., 2019; Tilg et al., 2020). Of particular interest is the proven links between gut microbiome health, intestinal dysbiosis and inflammation in people with CF (Rogers et al., 2010; Li and Somerset, 2014; Dhaliwal et al., 2015; Flass et al., 2015; Nielsen et al., 2016). This identified crosstalk occurring between the intestinal microbiota and the lungs has been termed the gut–lung axis, and there is growing interest in understanding how intestinal dysbiosis could potentially impact the progression and severity of airway disease in CF (**Figure 1**) (Marsland et al., 2015).

The neonatal period has been established as a critical stage in microbiota development and immune system maturation (Pattaroni et al., 2018). CFTR dysfunction has been shown to present as gut complications from birth that affect infant development, negatively impact the establishment of a beneficial gut and airway microbiota and consequently continue throughout adulthood (Bronstein et al., 1992; Li and Somerset, 2014; Leung et al., 2015; Boutin and Dalpke, 2017; Burke et al., 2017). Intestinal dysbiosis and inflammation observed from birth (Munck, 2014; Hoen et al., 2015) results in a decrease in beneficial gut commensals that could adversely affect the airway microbiota (Scanlan et al., 2012).

Evidence of the crosstalk which occurs between the intestinal and airway microbiota has been strengthened considerably by the discovery that both the gut (Ríos-Covián et al., 2016) and lungs (Mirković et al., 2015) are dominated by bacteria producing short-chain fatty acids (SCFA) and that those found in the lung are likely to originate from the gut. The mesenteric lymphatic system connects the gut with the lungs and it is possible this is the route by which bacteria or their metabolites could “seed” the airways; this could result in modulation of the immune response, as SCFAs have been shown to promote anti-inflammatory mechanisms and maintain intestinal homeostasis (Peng et al., 2009; Invernizzi et al., 2020). It is therefore conceivable that methods to combat microbial dysbiosis in one community could positively influence the other (Budden et al., 2017).

Targeted investigation and quantification of metabolites could provide better understanding of how the metabolic activity of the lung and gut microbiota affects respiratory health. Metabolomics offers the opportunity to validate and identify those metabolites and pathways of microbial origin, discern their interaction with the host and any potential role in inflammation and immune system development (Lee-Sarwar et al., 2020). Furthermore, the establishment of distinct microbiome and metabolome signatures offers the opportunity to advance a more personalized approach to patient diagnosis and treatment; for example, metabolomic profiles of exhaled breath condensate (EBC) have been considered as a potential prognostic biomarker for individuals with chronic respiratory disorders (Maniscalco et al., 2019) and for the early detection of pulmonary exacerbations in PWCF (Zang et al., 2017).

## Microbiota Diversity as an Indicator of Lung Inflammation

Inflammation of the airways is a common biological response to damage or infection and though largely beneficial it can play a key role in influencing the composition of the airway microbiome (Huffnagle et al., 2017). In the CF airways, increased secretion of proinflammatory mediators (e.g. IL-6 and IL-8) by potentially defective airway epithelium and immune cells leads to the disproportionate influx of neutrophils. Subsequent release of proteases, such as neutrophil elastase, damage structural lung proteins leading to an inevitable decline in pulmonary function.

Thus, the development of a range of microbiome-based molecular biomarkers would be a useful tool to monitor the onset or progression of airway disease, giving clinicians advanced warning of changing conditions and guiding therapeutic interventions. Rapid PCR diagnostics could be employed to determine the presence/absence of specific indicator organisms, such as known pathogens or established beneficial commensals, with a change in the load of either predicting the onset of deteriorating conditions (Harun et al., 2011; Boutin et al., 2018; Taylor et al., 2019). Pathogenic Proteobacteria possess potent inflammation enhancing pathogen-associated molecular patterns, such as lipopolysaccharides, and their association with inflammatory disorders is well known (Larsen et al., 2015; Rizzatti et al., 2017). For this reason, it has been suggested that this phylum could be used as an indicator of disease or marker of microbiota instability (Shin et al., 2015; Litvak et al., 2017). Likewise, messenger molecules involved in the host inflammatory response could also form the basis for further PCR targets (Eckrich et al., 2017).

As genomics technologies continue to advance, it is conceivable that monitoring of airway bacterial communities will become routine and so by utilizing either MGA or WGSS, the host’s respiratory microbiota could potentially function as a marker (Acosta et al., 2018; Sherrard and Bell, 2018). Comparing it to an idealized healthy benchmark may give advanced warning of unfavorable alterations in the composition of the microbiota, or potential “blooms” of pathogens, allowing for earlier intervention, especially if the patient is harboring slow growing pathogens or strains resistant to standard clinical culture (Harris et al., 2007; Woo et al., 2008). Recently Keravec et al. (2019) used a 16S MGA approach to identify prognostic biomarkers of *P. aeruginosa* infection in patients with CF and proposed representatives of the genus *Porphyromonas* as possible predictive markers.

## Microbiota Directed Therapeutic Approaches

If correctly exploited, the information generated by multi-omics investigations of the complete airway biome offers the potential to develop novel methods for the treatment of CF. Microbiome-based interventions offer greater scope for controlling health outcomes and although this field is in its infancy, options on how to leverage this untapped resource are discussed below.

### Antibiotic Regimen Optimization

One of the first studies to utilize culture-independent microbiota data in the selection of antimicrobial therapy for the treatment of pulmonary exacerbations in PWCF is the “Cystic Fibrosis Microbiome-determined Antibiotic Therapy Trial in Exacerbations: Results Stratified (CFMATTERS) Study” (Einarsson et al., 2017; Einarsson et al., 2019b). This study was designed to determine targeted antibacterial therapy compared to standard empirical therapy in the treatment of pulmonary exacerbations. Antibiotic treatment was selected based on the bacterial composition of a patient’s microbiota determined *via* NGS. Patients were randomized to either the control group where they received empirical antimicrobial therapy (ceftazidime and tobramycin/aztreonam) or the microbiome-directed treatment group where they received empirical therapy plus an additional antibiotic based on the top four most abundant taxa determined *via* NGS of a baseline sputum sample. However, there was no significant difference in the primary outcome, percentage change in recovery (post-exacerbation) FEV_1_ relative to the previous pre-exacerbation FEV_1_, between groups. Furthermore, in both the microbiome directed and empirical treatment arms, community composition appeared relatively stable over time, despite often showing a major disruption in community composition during the period of active antibiotic treatment. This highlights the need to further understand how bacterial community composition is affected in chronic respiratory diseases, as well as how a disruption or longer stability in the community composition impacts inflammatory processes within the airways of chronically infected PWCF.

### Enhanced Culturomics

Culture-independent metagenomics methods have proven expedient for generating vast quantities of data and accelerating our understanding of microbiomes. This has led to suggestions they could supplant traditional time-consuming culture techniques despite evidence that bacterial cultivability is much higher than previously thought (Kaeberlein et al., 2002). However, these methods can be disadvantaged by imperfect data analysis or experimental design (Bilen et al., 2018). Culturomics promises to bridge gaps in our knowledge by identifying unassigned sequences and uncovering new bacterial strains missed or overlooked by NGS (Lagier et al., 2012; Browne et al., 2016; Lagier et al., 2016) and provide more comprehensive data than metagenomics can alone (Dubourg et al., 2013). Using diverse and large-scale culture conditions, often complimented with MALDI-TOF and metagenomics, it has been possible to isolate and sequence previously uncultivable strains providing functional data to improve metagenomics. To more fully explore host-microbe interactions, complete the identification of microbiomes and test possible therapies, culturing pure isolates is still desirable (Lagier et al., 2015). Recent investigation of the CF lung by these methods demonstrated that not only could the majority of OTUs identified by amplicon sequencing be cultured but many more OTUs were described by culturomics than direct sequencing (Whelan et al., 2020).

### Pre-, Pro- and Syn-Biotic Therapies

The use of nutritional supplements containing substrates that stimulate the growth of beneficial microbes, live microorganisms, or combinations of both has been considered for some time as a treatment for intestinal dysbiosis (Vyas and Ranganathan, 2012; Coffey et al., 2018). The implication that microbiomes in anatomically distinct and distal sites, such as the gut and lungs, can communicate with each other presents an opportunity for direct and indirect targeted treatment of airway disease. Once such approach would be modulation of the intestinal microbiota in ways we know influence the airways beneficially, such as supporting the growth of gut commensals identified as indirectly enhancing alveolar macrophages and lung immune function (Clarke, 2014; Trompette et al., 2014; Martin et al., 2015). Moreover, directly transplanting bacteria into the lungs that increase diversity or seeding the airways with abiotic growth factors may improve respiratory health. Such therapies could possibly be used in addition to conventional therapy for treatment of chronic lung inflammation. However, they would require a high degree of personalization to ensure the combination of bacteria used produces the intended results and does not further exacerbate inflammation, a potential consequence if the bacterial constituents of the treatment are not normal members of the patients microbiota. Although limited in nature, human and murine investigations using prebiotics have shown promising effects on partially correcting intestinal dysbiosis and positively impacting colitis and ulcerative colitis symptoms (Hanai et al., 2004; Casellas et al., 2007; Koleva et al., 2012). Probiotic studies are much more varied and have focused principally on supplementation with *Lactobacillus* and *Bifidobacteria* spp. Symptom severity and improved lung function in children suffering from asthma was noted following administration of capsules containing *Lactobacillus gasseri* A5 (Chen et al., 2010) or a combination of *Lactobacillus acidophilus*, *Bifidobacterium bifidum* and *Lactobacillus delbrueckii* subsp. *Bulgaricus* (Gutkowski et al., 2010). However, after initially promising observations showing restoration of gut microbiota in children with CF when treated orally with *Lactobacillus* GG (Bruzzese et al., 2014), further investigation by the same group showed no significant clinical effect on respiratory outcomes (Bruzzese et al., 2018). The best known naturally occurring synbiotic is human breast milk (Martín et al., 2005) and the link between breastfeeding and lower incidence of asthma and associated airway inflammation is well documented (Oddy et al., 2002; Corey et al., 2019). Collectively, the use of prebiotic, probiotic or synbiotics to potentially suppress identified airway pathogens or reinstate beneficial taxa that have been eliminated during disease is promising. However substantial additional data, including *in vivo* studies, is required before it can be conclusively proven they positively impact the progression of CF chronic airway inflammation.

### Microbiome Derived Biomolecules

Genome mining of data acquired by WGSS can identify and characterize the biosynthetic gene clusters (BGCs) present within microbiomes that encode for bacterial natural products (BNPs), presenting an opportunity to discover unique biomolecules that could deliver new treatments for reducing inflammation and combating pathogen colonization (Vakhlu et al., 2008; Bachmann et al., 2014; Cheng et al., 2019). Multi-omics approaches can be used to analyze enzymes encoded by these gene clusters and any resulting products identified experimentally (Ziemert et al., 2016). This shift in focus from using culture-independent techniques to purely describe what constitutes a microbiota to what metabolic functions the microbiota demonstrate has revealed the vast extent of BGCs present and the drug-like products they encode for (Donia et al., 2014).

Given the complex communities and competition for resources that occurs within microbiomes, an extensive range of BNPs that exhibit narrow spectrum anti-microbial effects have been identified (Arnison et al., 2013). An opportunity to identify and develop microbiome-sourced antimicrobial compounds is an exciting possibility for the treatment of respiratory diseases. The use of natural products as therapies could reduce the use of broad-spectrum antibiotics and avoid associated complications such as dysbiosis and increased AMR. Lantibiotics have been found in both commensals such as *Staphylococcus epidermidis* (Velásquez et al., 2011) and human pathogens like *Enterococcus faecalis* (Sawa et al., 2012). Microcins are potent antibacterials derived exclusively from both commensal and pathogenic enterobacteria (Duquesne et al., 2007). TOMMs (thiazole/oxazole modified microcins) are related to microcins and display prolific functional diversity with over 300 TOMM BGCs identified to date (Melby et al., 2011). Bacterial pathways that exert anti-inflammatory responses could be inferred from gene mining of shotgun sequencing data. From this, BNPs that show beneficial immunomodulatory activity, could be identified and form the basis of new treatment opportunities.

### Modulate Microbiome Interactions

Deciphering the biochemical mechanisms between host cells and the microbiota offer novel avenues for therapy (Holmes et al., 2012) as it is now understood that the biotransformation of xenobiotics is commonly affected by the genetics of the host microbiome (Clayton et al., 2009; Wikoff et al., 2009) and they in turn can influence bacterial signaling and stress response pathways within the system (Maurice et al., 2013).

Metatransciptomics (Shakya et al., 2019) could be employed to examine the chemical roles bacterial members perform within the microbiome. This could provide specific information on bacterial metabolite usage, enzyme induction, regulation of inflammatory markers, secondary metabolism, the efficacy and unintended consequences of administered drugs and may reveal components of the microbiota that could be disrupted or selectively “drugged” (Wallace and Redinbo, 2013). Synthetic systems models are now being developed to study host-microbe interactions (Elzinga et al., 2019), an important step due to the growing appreciation that the gut microbiota and its metabolites can influence and modulate host immune function (Li and Somerset, 2018) and that these metabolites are present in distal organs such as the lungs (Schroeder and Bäckhed, 2016).

### Synthetic Bio-Delivery

An emerging field of therapeutic research is the use of synthetic engineering and the native microbiome itself for drug delivery and modulation of disease *via* either genetically modified bacteria (Nicaise et al., 2008; Mimee et al., 2016; Singhvi et al., 2018), vesicles (Chellappan et al., 2019) or phages (Nobrega et al., 2015). The latter has already shown promising results for the treatment of drug-resistant *M. abscessus* and *P. aeruginosa* infections in CF (Alemayehu et al., 2012; Dedrick et al., 2019; Law et al., 2019) and against dual-species biofilms formed by both *P. aeruginosa* and *S. aureus* (Tkhilaishvili et al., 2020). Deacon et al. (2015) enhanced the effectiveness of nanoparticle encapsulated tobramycin through DNase functionalization, improving CF sputum penetration. Understanding of the microbiome using enhanced culture-dependent and independent methods will be key to maximizing the advantages provided by synthetic engineering and its use as a therapy for respiratory disease.

## Future Directions

With the transformative potential that NGS technologies and metagenomics approaches promise, it is tempting to consider them a sole successor to all other current forms of testing and employ them wholesale in all areas of medicine and public health. However, considerable refinement and development of these sequencing technologies, their necessary downstream analytical pipelines, and investment in bioinformatic support is still required before widespread adoption into routine practice. Further studies utilizing these approaches are necessary to improve our understanding of the nature and composition of the lung microbiome, address the mechanisms by which microbe and host interact and ultimately the impact the respiratory microbiome in CF has on health and disease. More information resulting from such investigations will prove instrumental in improving molecular diagnostics and developing novel therapeutic approaches for the treatment of lung disease.

## Author Contributions

AL conceptualized and wrote the manuscript with support from GE. GE, DG, and MT provided consultation and critical revision of the manuscript. All authors contributed to the article and approved the submitted version.

## Funding

AL and GE are funded by the Innovative Medicines Initiative Joint Undertaking – European Union’s Seventh Framework Programme (FP7/2007–2013) and EFPIA companies in kind contribution (no. # 115721-1).

## Conflict of Interest

The authors declare that the research was conducted in the absence of any commercial or financial relationships that could be construed as a potential conflict of interest.
